# Stability of SF-36 profiles between 2007 and 2016: A study of 27,302 patients surgically treated for lumbar spine diseases

**DOI:** 10.1186/s12955-022-01999-7

**Published:** 2022-06-07

**Authors:** Anders Joelson, Freyr Gauti Sigmundsson, Jan Karlsson

**Affiliations:** 1 Department of Orthopedics, School of Medical Sciences and Orebro University Hospital, Orebro University, 70185 Orebro, Sweden; 2 Faculty of Medicine and Health, University Health Care Research Center, Orebro University, 70182 Orebro, Sweden

**Keywords:** Health-related Quality of life, Health profiles, Stability, Lumbar spine diseases, SF-36

## Abstract

**Background:**

Previous studies have shown that patients with different lumbar spine diseases report different SF-36 profiles, but data on the stability of the SF-36 profiles are limited. The primary aim of the current study was to evaluate the stability of the SF-36 profile for lumbar spine diseases.

**Methods:**

Patients, surgically treated between 2007 and 2016 for three lumbar spine diseases, lumbar spinal stenosis (LSS) with degenerative spondylolisthesis (DS), LSS without DS, and lumbar disk herniations (LDH), were identified in the Swedish spine register. Preoperative and 1 year postoperative SF-36 data for a total of 27,302 procedures were available for analysis. The stability of the SF-36 profiles over the 10-year period was evaluated using graphical exploration, linear regression, difference in means, and 95% confidence intervals. The responsiveness of the SF-36 domains to surgical treatment was evaluated using the standardized response mean (SRM).

**Results:**

LSS and LDH have different SF-36 profiles. LSS with DS and LSS without DS have similar SF-36 profiles. The preoperative and the 1 year postoperative SF-36 profiles were stable from 2007 to 2016 for all three diagnoses. There were no major changes in the effect size of change (SRM) during the study period for all three diagnoses. For LSS with DS, the number of fusions peaked in 2010 and then decreased. The postoperative SF-36 profiles for LSS with DS were unaffected by changes in surgical treatment trends.

**Conclusions:**

Patients with lumbar spinal stenosis and lumbar disk herniations have different SF-36 profiles. Concomitant degenerative spondylolisthesis had no impact on the SF-36 profile of lumbar spinal stenosis. Adding fusion to the decompression did not alter the postoperative SF-36 profile of lumbar spinal stenosis. The SF-36 health profiles are stable from a 10 years perspective.

**Supplementary Information:**

The online version contains supplementary material available at 10.1186/s12955-022-01999-7.

## Introduction

Since its introduction in 1992, the SF-36 health survey [1] has been used worldwide in clinical trials, population studies, and in clinical settings for the assessment of health-related quality of life (HRQoL). The instrument describes HRQoL as a health profile with eight domains. Previous studies have shown that patients with different lumbar spine diseases report different SF-36 profiles [2]. This is not surprising since patients with different diagnoses experience different problems, for example, lumbar disk herniation (LDH) often present with radiculopathy whereas neurogenic claudication is a hallmark symptom in lumbar spinal stenosis (LSS). Surprisingly few studies, however, have investigated the stability of the SF-36 profiles. Our literature review identified only one relevant study [3] so there is a clear knowledge gap on the topic today. The primary aim of the current study was to evaluate the stability of the SF-36 profiles in lumbar spine diseases.

Trends in surgical treatment of lumbar spine diseases may change over time. For the US population, Bae et al. [4] reported an increase in simple fusions for lumbar spinal stenosis (LSS) from 2004 to 2009, while the decompressions decreased during the same time period. In another US study, Al Jammal et al. [5] found an increasing number of simple fusions for LSS without coexisting scoliosis between 2010 and 2014 whereas the decompression rate decreased over the same time period. The secondary aim of the current study was to evaluate whether the long-term SF-36 profiles were affected by changes in the surgical treatment of lumbar spine diseases.

## Methods

### Study design

The present study was a register study, with prospectively collected longitudinal data from the national Swedish spine register, Swespine.

### The national Swedish spine register, Swespine

The national Swedish spine register (Swespine) was launched at Lund university hospital in southern Sweden in 1992 and became national in 1998. The coverage is 90% of the 60 spine units in Sweden and the 1 year follow-up rate is 70–75% [6]. The register includes data on diagnoses, surgical procedures, complications, and patient-reported outcome measures (PROMs). The surgeon is responsible for submitting data about the surgery whereas the patient submits background data and completes the PROM forms. The Swespine office organizes the follow-up and the surgeons are not involved.

### Measures

The SF-36 is an 8-dimensional, 36-item, self-administered HRQoL instrument for the assessment of general HRQoL [1]. We used the Swedish translation of SF-36 version 1 in our study [7]. 35 items (not item 2) are used to assess 8 dimensions of health. The dimensions (domains) are: physical functioning (PF), role limitation due to physical problems (RP), bodily pain (BP), general health (GH), vitality (VT), social functioning (SF), role limitations due to emotional problems (RE), and mental health (MH). The results are presented as a health profile, where the score for each domain ranges from 0 to 100 (0 being the worst and 100 the best).

### Patient data set

Patients, who were surgically treated between 2007 and 2016 for three lumbar spine diseases, lumbar spinal stenosis with more than 3 mm degenerative spondylolisthesis (LSS with DS), LSS with less than or equal to 3 mm DS (LSS without DS), and lumbar disk herniation (LDH), were identified in the Swedish spine register. A total of 50,705 surgical procedures (decompression only, decompression and instrumented fusion, diskectomy and microdiskectomy) for treatment of the three lumbar spine diseases between 2007 and 2016 are included in the register. Preoperative or 1-year postoperative SF-36 data were incomplete for 23,403 (46%) of the procedures which gave 27,302 procedures eligible for analysis. Baseline patient characteristics are shown in Table 1. The characteristics of the excluded patients are presented in Additional file 1: Table S1.

**Table 1:** Characteristics of the study population

	LSS with DS	LSS without DS	LDH
n	4827	13,543	8932
Age, mean (SD)	67.3 (9.27)	67.6 (10.1)	45.3 (13.3)
BMI, mean (SD)	27.1 (4.33)	27.7 (4.14)	26.2 (4.14)
Women, n (%)	3444 (71.3)	6319 (46.7)	4109 (46)

### Statistics

Data are presented as mean and standard deviation (SD) and/or 95% confidence intervals (CIs). Bootstrapping was used to calculate the CIs [8]. Trends in SF-36 outcome were analyzed with linear regression. Standardized response means (SRMs) for paired data, that is, the difference in means divided by the standard deviation of the difference, were used to evaluate the effect sizes of change between preoperative and postoperative SF-36 data [9]. The SRMs were interpreted as follows: less than 0.20 no effect, 0.20 to 0.49 small effect, 0.50 to 0.79 moderate effect, greater than or equal to 0.80 large effect [9].

## Results

**Fig. 1:**
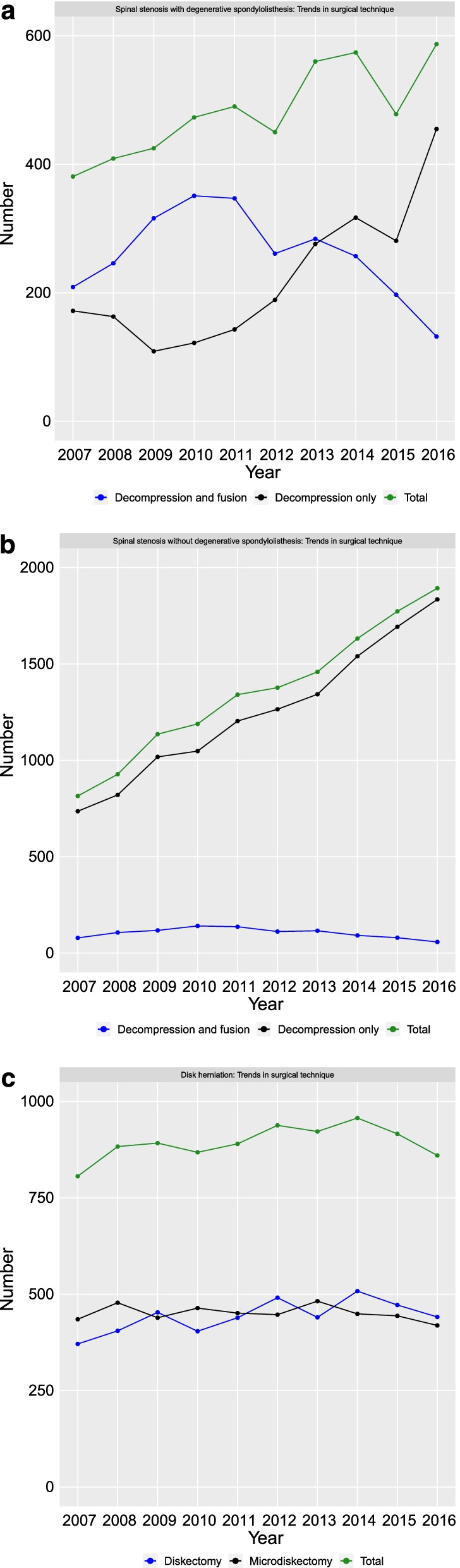
**a**–**c** Trends in surgical treatment for patients with lumbar spinal stenosis with degenerative spondylolisthesis, lumbar spinal stenosis without degenerative spondylolisthesis, and lumbar disk herniations between 2007 and 2016

The Swedish nationwide trends in the surgical management of LSS with DS, LSS without DS, and LDH between 2007 and 2016 are illustrated in Figs. 1a–c. There were an increasing number of surgeries for LSS during the study period and a constant number of surgeries for LDH. For LSS with DS, the number of fusions peaked in 2010 and then decreased by more than 50% whereas the number of patients treated with decompression only increased by more than 100% during the study period.

**Fig. 2:**
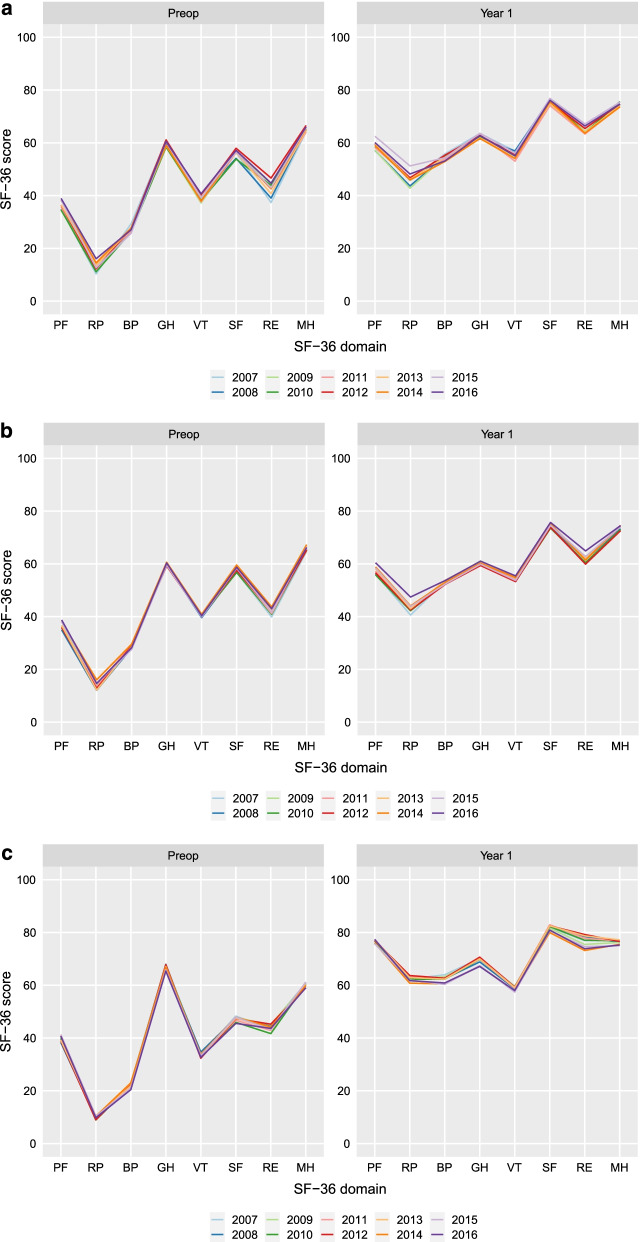
**a** SF-36 profiles preoperatively and 1-year postoperatively for patients treated for lumbar spinal stenosis with degenerative spondylolisthesis between 2007 and 2016. **b** SF-36 profiles preoperatively and 1 year postoperatively for patients treated for lumbar spinal stenosis without degenerative spondylolisthesis between 2007 and 2016. **c** SF-36 profiles preoperatively and 1 year postoperatively for patients treated for lumbar disk herniation between 2007 and 2016

Figures 2a–c and Additional file 1: Tables S2a–c and S3 illustrate the SF-36 profiles before and 1 year after surgery for the three diagnoses between 2007 and 2016. Patients with LSS and LDH had different preoperative SF-36 profiles. In addition, the 1 year postoperative SF-36 profiles differed between LSS and LDH. In contrast, LSS with DS and LSS without DS had similar SF-36 profiles. The 1 year postoperative SF-36 profiles were similar for LSS with DS and LSS without DS. The shapes of the preoperative and 1-year postoperative SF-36 profiles were stable during the 10 years time period (Fig. 2a–c and Additional file 1: Tables S2a–c). There were no major changes in the effect size of change (SRM) during the study period (Additional file 1: Tables S2a–c).

Additional file 1: Figures S1a–c illustrate the trends in SF-36 scores over time. Linear regression analysis of these trends are presented in Additional file 1: Tables S4a–c. The 95% CIs of the regression line slopes included zero (i.e. no statistically significant change over time) in 71% (17/24) of the preoperative regression lines, in 54% (13/24) of the 1-year postoperative regression lines, and in 96% (23/24) of the 1-year postoperative preoperative difference regression lines.

## Discussion

In the present study, we evaluated the stability of the SF-36 profiles for three lumbar spine diseases. We found that both the preoperative and 1 year postoperative SF-36 profiles were stable between 2007 and 2016. Few studies have evaluated the stability of the SF-36 profiles. Jacobsen et al. [3] collected Norwegian population-based SF-36 data at three time points (1996, 2002, and 2015) and found that the profiles remained relatively stable despite significant societal changes in the Norwegian population (for example, higher education level and longer life expectancy). The findings of our study extend the Norwegian results to the subgroup of patients with lumbar spine diseases.

The findings of our study can be interpreted in terms of sensitivity and responsiveness of the SF-36 health survey. Sensitivity is the ability of measurements to detect differences between patients or groups of patients [9]. Our data verified that patients with different lumbar spine diseases report different SF-36 profiles and that these profiles are stable from a 10 years perspective. This means that the SF-36 can separate groups of patients with different diagnoses, implying that the SF-36 health survey is sensitive to disease-specific differences in lumbar spine conditions. However, parts of the differences in SF-36 profiles, most probably, are explained by reasons other than disease-specific factors e.g. sociodemographic differences like age or gender. Responsiveness is the ability to detect changes when a patient improves or deteriorates [9]. Our measurements of the effect size of change (SRM) were stable over the course of 10 years, with substantial differences in preoperative and postoperative health profiles. Consequently, the SF-36 provides stable measurements of responsiveness to surgical treatment of spine diseases. Furthermore, we show that the SF-36, over a 10 years period, produces very similar health profiles, for different respondents with the same diagnosis, suggesting that SF-36 provides stable measurements of health.

Our regression analyses showed that the majority of the regression line slope CIs included zero, i.e. no statistically significant change over time (Additional file 1: Tables S4a–c). Of the significant trends, the maximum mean slope was 0.55 (RP for LSS with DS year 1) which means that the change over a course of 10 years was 5.5 points on the 0 to 100 scale. This is far below previously reported values of the minimal important change (MIC) [10].

We found that LSS and LDH had different preoperative SF-36 profiles. In addition, the 1 year postoperative SF-36 profiles differed between LSS and LDH. This confirms the findings of Hansson et al. [11] and Zanoli et al. [2] that patients with different lumbar spine diseases report different SF-36 profiles. Our study contributes to the literature by demonstrating that these profiles are reproducible and stable over a 10 years period.

We found that all domains except the SF-36 GH scale were improved 1 year after surgery from an effect size of change perspective. This confirms the limited responsiveness of the GH scale as shown in previous spine studies [11–13]. In general, the effect sizes of 1 year change (SRM) were larger for LDH than for LSS. Notably, all accumulated 1 year postoperative values were below the norms for general Swedish population (Additional file 1: Table S3).

Interestingly, the SF-36 profiles were stable for LSS with DS although there was a marked shift from decompression and fusion to decompression only from 2007 to 2016. This means that, interpreted from an SF-36 perspective, decompression only is not inferior to decompression and fusion for the treatment of LSS with DS. Controversy remains concerning decompression only versus decompression and fusion for LSS with DS. Two register studies [14, 15] and two randomized controlled trials [16, 17] showed no clinically meaningful differences between decompression only and decompression and fusion for LSS with DS. Another randomized controlled trial, however, concluded that decompression and fusion was associated with slightly better and clinically meaningful improvements in physical HRQoL than decompression only [18]. The studies by Försth et al. [16] and Austevoll et al. [17] used the Oswestry disability index (ODI) [19] as the primary outcome measure, whereas the study of Ghogawala et al. [18] used the SF-36 physical component summary score (PCS) [20] as the primary outcome measure. One could hypothesize that a more fine-grained measure, such as the SF-36 health profile could more precisely identify differences between the different treatment options for LSS with DS than a single-point outcome measures such as ODI and PCS. Our study, however, could not identify any changes in SF-36 health profiles during the time period when the surgical treatment of LSS with DS shifted from decompression and fusion to decompression only.

In contrast to our finding of a marked shift in the surgical treatment of LSS with DS, Al Jammal et al. [5], based on the US National Inpatient Sample Database, reported an increasing number of simple fusions for LSS without coexisting scoliosis from 2010 to 2014, whereas the decompression rate decreased during the same time period. On the other hand, the Finnish National Hospital Discharge Register data of Mäntymäki et al. [21], suggested that the number of fusions peaked in 2014, while the decompressions increased steadily. The Swedish shift in surgical preference for the treatment of LSS with DS observed in our data may have several causes. Possibly, the Swedish LSS register study [14] followed by the Swedish randomized controlled trial [16] may have had a major impact on lumbar fusion indications in Sweden, as preliminary results early were made available to Swedish spine surgeons. Furthermore, because of the relatively small number of surgical procedures in Sweden, policy changes at a few high-volume units or changes in the preferences of individual high-volume surgeons, may have a large impact on surgical trends, including the number of fusions.

Based on data from the Finnish National Hospital Discharge Register, Mäntymäki et al. [21] report a decreasing rate of LDH surgery procedures from 1997 to 2006 and a stable rate between 2007 and 2016. Similarly, we report no major change in the number of yearly LDH surgeries between 2007 and 2016.

Our findings should be evaluated in the light of several limitations. First, we recognize the inherent limitations of register data, such as a lack of confounder information, missing data, or unknown data quality [22]. Second, the data were limited to spine surgery patients, that is, persons with problems mainly related to the musculoskeletal system. Third, information about co-morbidities that might affect heath profiles was lacking. Fourth, data was incomplete for 46% of the procedures. Fifth, there were differences in baseline characteristics between included and excluded patients (mean age difference 1.0 to 1.9 years, mean BMI difference 0.1 to 0.4 kg/m^2^, and gender difference 0.3 to 2.9 percent). The differences are small and are likely to have only limited clinical relevance. Sixth, we did not adjust our data for possible confounders and is thus possible that the patient case mix differed between years, which might perhaps mask a possible yearly effect. Seventh, no correction for multiple testing was made which increases the probability for type I errors, especially in Additional file 1: Tables S2a–c. The lack of statistically significant findings may be a consequence of type II errors.

## Conclusions

Patients with lumbar spinal stenosis and lumbar disk herniations have different SF-36 profiles. Concomitant degenerative spondylolisthesis had no impact on the SF-36 profile of patients with lumbar spinal stenosis. Adding fusion to the decompression did not alter the SF-36 profile of lumbar spinal stenosis. The SF-36 health profiles are stable from a 10 years perspective.

## Supplementary Information

**Additional file 1:** Supplementary Tables and Figures.

## Data Availability

Data are available from the national Swedish spine register (Swespine) after approval by the Swedish Ethical Review Authority and according to regulations in the General Data Protection Regulation and the Swedish Patient Data Act.

## Abbreviations

CI: Confidence interval
DS: Degenerative spondylolisthesis
HRQoL: Health-related quality of life
LDH: Lumbar disk herniation
LSS: Lumbar spinal stenosis
SD: Standard deviation
SRM: Standardized response mean

## References

[1] Ware JE, Sherbourne CD (1992). The MOS 36-item short-form health survey (SF-36). I. Conceptual framework and item selection. Med Care.

[2] Zanoli G, Jönsson B, Strömqvist B (2006). SF-36 scores in degenerative lumbar spine disorders: analysis of prospective data from 451 patients. Acta Orthop.

[3] Jacobsen EL, Bye A, Aass N, Fosså SD, Grotmol KS, Kaasa S, Loge JH, Moum T, Hjermstad MJ (2018). Norwegian reference values for the short-form health survey 36: Development over time. Qual Life Res.

[4] Bae HW, Rajaee SS, Kanim LE (2013). Nationwide trends in the surgical management of lumbar spinal stenosis. Spine (Phila Pa 1976).

[5] Al Jammal OM, Delavar A, Maguire KR, Hirshman BR, Wali AR, Kazzaz M, Pham MH (2019). National trends in the surgical management of lumbar spinal stenosis in adult spinal deformity patients. Spine (Phila Pa 1976).

[6] Strömqvist B, Fritzell P, Hägg O, Jönsson B, Sandén B (2013). Swedish society of spinal surgeons. Swespine: the Swedish spine register: the 2012 report. Eur Spine J.

[7] Sullivan M, Karlsson J, Ware JE (1995). The Swedish SF-36 health survey-I. Evaluation of data quality, scaling assumptions, reliability and construct validity across general populations in Sweden. Soc Sci Med.

[8] Bland JM, Altman DG (2015). Statistics notes: bootstrap resampling methods. BMJ.

[9] Fayers PM, Machin D (2016). Quality of life: the assessment, analysis and reporting of patient-reported outcomes.

[10] Kosinski M, Zhao SZ, Dedhiya S, Osterhaus JT, Ware JE (2000). Determining minimallyimportant changes in generic and disease-specific health-related quality of life questionnaires in clinical trials of rheumatoid arthritis. Arthritis Rheum.

[11] Hansson T, Hansson E, Malchau H (2008). Utility of spine surgery: a comparison of common elective orthopaedic surgical procedures. Spine (Phila Pa 1976).

[12] Carreon LY, Berven SH, Djurasovic M, Bratcher KR, Glassman SD (2013). The discriminative properties of the SF-6D compared with the SF-36 and ODI. Spine (Phila Pa 1976).

[13] Joelson A, Sigmundsson FG, Karlsson J (2021). Responsiveness of the SF-36 general health domain: observations from 14883 spine surgery procedures. Qual Life Res.

[14] Försth P, Michaëlsson K, Sandén B (2013). Does fusion improve the outcome after decompressive surgery for lumbar spinal stenosis?: a two-year follow-up study involving 5390 patients. Bone Joint J.

[15] Austevoll IM, Gjestad R, Solberg T, Storheim K, Brox JI, Hermansen E, Rekeland F, Indrekvam K, Hellum C (2020). Comparative effectiveness of microdecompression alone vs decompression plus instrumented fusion in lumbar degenerative spondylolisthesis. JAMA Netw Open.

[16] Försth P, Ólafsson G, Carlsson T, Frost A, Borgström F, Fritzell P, Öhagen P, Michaëlsson K, Sandén B (2016). A randomized, controlled trial of fusion surgery for lumbar spinal stenosis. N Engl J Med.

[17] Austevoll IM, Hermansen E, Fagerland MW, Storheim K, Brox JI, Solberg T, Rekeland F, Franssen E, Weber C, Brisby H, Grundnes O, Algaard KRH, Böker T, Banitalebi H, Indrekvam K, Hellum C, Nordsten DS (2021). Decompression with or without fusion in degenerative lumbar spondylolisthesis. N Engl J Med.

[18] Ghogawala Z, Dziura J, Butler WE, Dai F, Terrin N, Magge SN, Coumans JVCE, Harrington JF, Amin-Hanjani S, Schwartz JS, Sonntag VKH, Barker FG, Benzel EC (2016). Laminectomy plus fusion versus laminectomy alone for lumbar spondylolisthesis. N Engl J Med.

[19] Fairbank JC, Couper J, Davies JB, O’Brien JP (1980). The Oswestry low back pain disability questionnaire. Physiotherapy.

[20] Ware JE, Kosinski M, Keller SD (1994). SF-36 physical and mental health summary scales: a user’s manual.

[21] Mäntymäki H, Ponkilainen VT, Huttunen TT, Mattila VM (2021). Regional variations in lumbar spine surgery in Finland. Arch Orthop Trauma Surg.

[22] Thygesen LC, Ersbøll AK (2014). When the entire population is the sample: strengths and limitations in register-based epidemiology. Eur J Epidemiol.

