# Supplementary material for: Stability of SF-36 profiles between 2007 and 2016: A study of 27,302 patients surgically treated for lumbar spine diseases
Source: Health Qual Life Outcomes. 2022 Jun 7;20:92. doi: 10.1186/s12955-022-01999-7 (PMC9172105; doi:10.1186/s12955-022-01999-7)
Supplement: Supplementary file 1 — Additional file 1: Supplementary Tables and Figures. [file 12955_2022_1999_MOESM1_ESM.pdf]

Table S1 Characteristics of excluded patients.

	LSS with DS	LSS without DS	LDH
**n**	3292	10966	9145
**Age, mean (SD)**	68.8 (10.1)	68.6 (11.5)	43.4 (13.4)
**BMI, mean (SD)**	27.5 (4.4)	27.8 (4.3)	26.6 (4.4)
**Women, n (%)**	2358 (71.6)	5551 (50.6)	3937 (43.1)

Table S2a Preoperative and 1-year postoperative SF-36 data (mean and 95% CIs) for patients treated surgically for LSS with DS from 2007 to 2016.

		PF	RP	BP	GH	VT	SF	RE	MH
2007 (n=381)	**preop**	35 (33;37)	10 (7.9;13)	29 (28;31)	61 (59;63)	38 (36;41)	56 (54;59)	37 (33;42)	64 (62;66)
	**year 1**	59 (57;62)	46 (41;50)	56 (53;58)	64 (61;66)	57 (54;59)	77 (74;79)	64 (60;68)	75 (73;77)
	**diff**	24 (22;27)	35 (31;40)	26 (24;29)	2.5 (0.59;4.3)	18 (16;21)	20 (17;23)	26 (22;31)	10 (8.3;13)
	**srm**	0.98 (0.86;1.1)	0.82 (0.7;0.93)	0.99 (0.87;1.1)	0.13 (0.034;0.24)	0.75 (0.64;0.87)	0.73 (0.62;0.85)	0.55 (0.44;0.66)	0.48 (0.38;0.59)
2008 (n=409)	**preop**	35 (33;37)	12 (10;15)	27 (25;28)	59 (57;61)	40 (38;42)	54 (51;57)	39 (34;43)	65 (63;67)
	**year 1**	57 (55;59)	44 (40;48)	55 (52;57)	62 (59;64)	57 (54;60)	76 (74;79)	64 (59;68)	75 (72;77)
	**diff**	22 (20;25)	31 (27;35)	28 (25;31)	2.2 (0.19;4.1)	17 (15;19)	22 (19;25)	25 (20;30)	9.6 (7.5;12)
	**srm**	0.82 (0.71;0.94)	0.73 (0.62;0.84)	1.1 (0.93;1.2)	0.11 (0.013;0.21)	0.71 (0.6;0.82)	0.73 (0.62;0.84)	0.48 (0.38;0.58)	0.45 (0.35;0.55)
2009 (n=425)	**preop**	34 (32;36)	13 (10;15)	27 (26;28)	58 (56;60)	37 (35;39)	56 (54;59)	45 (40;48)	65 (63;67)
	**year 1**	57 (55;60)	43 (39;47)	55 (52;57)	62 (60;64)	55 (52;57)	76 (73;78)	64 (60;69)	75 (72;77)
	**diff**	23 (21;25)	30 (26;35)	28 (26;30)	3.3 (1.5;5.2)	18 (15;20)	20 (17;22)	20 (15;25)	9.8 (7.7;12)
	**srm**	1 (0.88;1.1)	0.67 (0.56;0.77)	1.1 (0.99;1.2)	0.17 (0.073;0.26)	0.69 (0.59;0.8)	0.7 (0.59;0.81)	0.4 (0.3;0.5)	0.45 (0.35;0.55)
2010 (n=473)	**preop**	35 (33;36)	11 (8.9;13)	26 (25;28)	58 (57;60)	38 (36;40)	54 (51;56)	44 (40;48)	65 (63;67)
	**year 1**	58 (56;61)	46 (42;50)	55 (52;57)	63 (60;65)	55 (53;57)	77 (74;79)	64 (60;68)	76 (74;77)
	**diff**	24 (21;26)	35 (31;39)	29 (26;31)	4.1 (2.5;5.8)	17 (15;19)	23 (20;25)	20 (16;24)	11 (8.7;12)
	**srm**	0.95 (0.84;1.1)	0.81 (0.71;0.91)	1.1 (0.95;1.2)	0.22 (0.13;0.31)	0.7 (0.6;0.8)	0.83 (0.72;0.93)	0.43 (0.34;0.53)	0.55 (0.45;0.64)
2011 (n=490)	**preop**	36 (34;37)	14 (12;17)	26 (25;28)	60 (59;62)	38 (36;40)	56 (54;59)	43 (39;47)	64 (62;66)
	**year 1**	58 (56;61)	47 (43;51)	54 (51;56)	63 (61;65)	53 (51;55)	74 (71;77)	63 (59;67)	74 (72;76)
	**diff**	23 (20;25)	33 (29;37)	27 (25;30)	2.7 (0.93;4.4)	15 (13;17)	18 (15;21)	21 (16;25)	9.8 (8.1;12)
	**srm**	0.96 (0.86;1.1)	0.72 (0.62;0.82)	1 (0.92;1.1)	0.14 (0.052;0.23)	0.66 (0.56;0.75)	0.6 (0.51;0.7)	0.41 (0.32;0.5)	0.5 (0.41;0.6)
2012 (n=450)	**preop**	36 (34;38)	12 (9.8;15)	28 (26;29)	61 (59;63)	40 (38;42)	58 (55;61)	47 (42;51)	67 (65;68)
	**year 1**	59 (57;62)	47 (43;51)	55 (53;58)	63 (61;65)	55 (53;58)	75 (73;78)	65 (62;69)	75 (73;77)
	**diff**	23 (21;25)	34 (30;39)	28 (25;30)	1.6 (-0.1;3.2)	15 (13;18)	18 (15;20)	19 (14;24)	8.1 (6.2;9.9)
	**srm**	0.93 (0.82;1)	0.75 (0.65;0.86)	1 (0.93;1.2)	0.09 (-0.0022;0.18)	0.67 (0.56;0.77)	0.63 (0.53;0.73)	0.36 (0.26;0.45)	0.41 (0.31;0.51)
2013 (n=560)	**preop**	37 (35;38)	14 (11;16)	28 (26;29)	59 (57;60)	39 (38;41)	56 (54;59)	41 (37;44)	65 (63;66)
	**year 1**	60 (58;62)	46 (42;50)	55 (52;57)	62 (60;63)	55 (53;57)	75 (73;77)	64 (60;68)	74 (72;76)
	**diff**	23 (21;25)	33 (29;36)	27 (25;29)	3 (1.3;4.6)	16 (14;18)	18 (16;21)	23 (19;27)	9.1 (7.5;11)
	**srm**	0.95 (0.85;1)	0.69 (0.6;0.78)	1 (0.9;1.1)	0.14 (0.06;0.23)	0.64 (0.54;0.73)	0.63 (0.54;0.72)	0.48 (0.39;0.56)	0.46 (0.37;0.54)
2014 (n=574)	**preop**	38 (36;40)	15 (12;17)	28 (26;29)	59 (58;61)	38 (36;40)	57 (54;59)	43 (39;46)	65 (64;67)
	**year 1**	59 (57;61)	46 (42;49)	53 (51;55)	62 (60;63)	54 (52;56)	76 (73;78)	64 (60;67)	74 (72;75)
	**diff**	21 (19;23)	31 (27;35)	25 (23;27)	2.6 (1.1;4.1)	16 (14;18)	19 (17;22)	21 (17;25)	8.3 (6.6;9.9)
	**srm**	0.84 (0.75;0.94)	0.68 (0.59;0.77)	0.96 (0.86;1.1)	0.13 (0.051;0.22)	0.68 (0.59;0.77)	0.66 (0.57;0.75)	0.41 (0.32;0.49)	0.41 (0.33;0.5)
2015 (n=478)	**preop**	38 (37;40)	13 (10;15)	26 (25;27)	60 (59;62)	39 (37;41)	57 (54;59)	43 (39;47)	66 (64;67)
	**year 1**	62 (60;65)	51 (48;55)	54 (52;57)	64 (62;65)	56 (54;58)	77 (75;79)	67 (64;71)	75 (74;77)
	**diff**	24 (22;26)	39 (35;42)	29 (26;31)	3.2 (1.5;5)	17 (15;19)	20 (17;23)	24 (19;29)	9.7 (7.9;12)
	**srm**	0.98 (0.87;1.1)	0.86 (0.75;0.96)	1.1 (0.94;1.2)	0.17 (0.078;0.26)	0.7 (0.6;0.8)	0.67 (0.57;0.77)	0.46 (0.36;0.55)	0.46 (0.36;0.55)
2016 (n=587)	**preop**	39 (37;41)	16 (14;19)	27 (26;28)	61 (59;62)	41 (39;42)	57 (55;59)	45 (41;48)	66 (65;68)
	**year 1**	60 (58;62)	48 (45;52)	53 (51;55)	63 (61;65)	55 (53;57)	76 (74;78)	66 (63;70)	75 (73;76)
	**diff**	21 (19;23)	32 (29;36)	26 (24;28)	2.2 (0.67;3.8)	14 (12;16)	19 (17;21)	22 (18;26)	8.6 (7;10)
	**srm**	0.88 (0.79;0.98)	0.73 (0.64;0.82)	0.99 (0.89;1.1)	0.12 (0.035;0.2)	0.6 (0.51;0.69)	0.68 (0.59;0.77)	0.45 (0.37;0.53)	0.42 (0.34;0.51)

Table S2b Preoperative and 1-year postoperative SF-36 data (mean and 95% CIs) for patients treated surgically for LSS without DS from 2007 to 2016.

		PF	RP	BP	GH	VT	SF	RE	MH
2007 (n=815)	**preop**	35 (34;37)	13 (11;15)	29 (28;31)	59 (58;61)	40 (39;42)	57 (55;59)	40 (37;43)	65 (63;66)
	**year 1**	56 (54;58)	41 (37;44)	54 (52;55)	59 (58;61)	54 (53;56)	74 (72;76)	60 (57;63)	74 (72;75)
	**diff**	21 (19;23)	28 (25;31)	24 (22;26)	0.15 (-1.2;1.6)	14 (12;16)	17 (15;19)	20 (17;23)	9.2 (7.8;11)
	**srm**	0.85 (0.77;0.93)	0.64 (0.56;0.71)	0.94 (0.85;1)	0.01 (-0.061;0.076)	0.58 (0.5;0.65)	0.62 (0.54;0.69)	0.4 (0.33;0.47)	0.46 (0.39;0.54)
2008 (n=928)	**preop**	35 (34;36)	12 (11;14)	28 (27;29)	60 (59;61)	40 (38;41)	57 (55;59)	42 (39;45)	66 (64;67)
	**year 1**	56 (55;58)	43 (40;45)	53 (52;55)	60 (59;62)	53 (52;55)	74 (72;76)	62 (59;64)	73 (72;75)
	**diff**	21 (20;23)	30 (28;33)	25 (24;27)	0.18 (-0.98;1.5)	14 (12;15)	17 (15;19)	19 (16;22)	7.7 (6.3;9)
	**srm**	0.86 (0.79;0.94)	0.68 (0.61;0.75)	0.93 (0.86;1)	0.01 (-0.055;0.07)	0.59 (0.52;0.66)	0.57 (0.5;0.64)	0.4 (0.34;0.47)	0.37 (0.31;0.44)
2009 (n=1136)	**preop**	37 (36;38)	14 (12;16)	29 (28;30)	60 (59;62)	40 (39;42)	58 (56;60)	44 (41;46)	65 (64;66)
	**year 1**	57 (56;59)	42 (40;45)	53 (52;55)	61 (60;62)	55 (53;56)	75 (73;76)	63 (60;65)	74 (73;75)
	**diff**	20 (19;22)	28 (26;31)	24 (23;26)	0.72 (-0.41;1.9)	14 (13;16)	17 (15;18)	19 (16;22)	9.2 (8.1;10)
	**srm**	0.81 (0.75;0.88)	0.63 (0.57;0.69)	0.93 (0.86;1)	0.036 (-0.02;0.094)	0.58 (0.52;0.65)	0.59 (0.53;0.65)	0.39 (0.33;0.45)	0.46 (0.4;0.52)
2010 (n=1189)	**preop**	36 (35;37)	13 (12;15)	29 (28;30)	60 (59;62)	40 (39;41)	57 (55;58)	41 (38;43)	65 (64;67)
	**year 1**	56 (54;57)	42 (40;45)	53 (51;54)	60 (59;62)	53 (52;55)	74 (72;75)	60 (58;63)	73 (72;74)
	**diff**	20 (19;21)	29 (26;31)	23 (22;25)	-0.056 (-1.1;1)	13 (12;14)	17 (15;18)	20 (17;22)	7.6 (6.5;8.8)
	**srm**	0.8 (0.73;0.86)	0.64 (0.57;0.7)	0.87 (0.8;0.93)	-0.003 (-0.06;0.05)	0.56 (0.5;0.62)	0.58 (0.52;0.64)	0.39 (0.33;0.45)	0.38 (0.32;0.44)
2011 (n=1341)	**preop**	36 (35;37)	13 (12;15)	30 (29;30)	60 (59;61)	40 (39;41)	58 (56;59)	41 (39;44)	66 (65;67)
	**year 1**	57 (56;59)	43 (41;45)	53 (51;54)	61 (60;62)	54 (53;55)	75 (73;76)	62 (59;64)	74 (73;75)
	**diff**	21 (20;22)	30 (27;32)	23 (22;25)	0.61 (-0.46;1.7)	14 (13;15)	17 (16;19)	20 (18;23)	8.5 (7.4;9.6)
	**srm**	0.85 (0.79;0.91)	0.67 (0.61;0.73)	0.9 (0.84;0.96)	0.03 (-0.023;0.084)	0.57 (0.52;0.63)	0.59 (0.53;0.65)	0.4 (0.34;0.45)	0.4 (0.34;0.45)
2012 (n=1377)	**preop**	36 (35;37)	13 (11;14)	29 (28;30)	59 (58;60)	40 (39;41)	58 (56;59)	42 (40;44)	65 (64;66)
	**year 1**	57 (55;58)	43 (40;45)	52 (51;54)	59 (58;61)	53 (52;55)	74 (72;75)	60 (58;62)	73 (71;74)
	**diff**	21 (19;22)	30 (28;32)	23 (22;25)	0.12 (-0.89;1.2)	13 (12;14)	16 (15;18)	18 (15;21)	7.4 (6.3;8.4)
	**srm**	0.83 (0.77;0.89)	0.67 (0.62;0.73)	0.9 (0.84;0.96)	0.006 (-0.05;0.06)	0.56 (0.5;0.62)	0.56 (0.5;0.61)	0.35 (0.3;0.41)	0.35 (0.3;0.41)
2013 (n=1459)	**preop**	37 (36;38)	12 (11;14)	29 (28;29)	60 (59;61)	41 (40;42)	58 (57;60)	42 (40;45)	67 (66;68)
	**year 1**	58 (57;59)	43 (41;45)	54 (52;55)	61 (60;62)	55 (54;56)	75 (73;76)	62 (60;64)	74 (73;75)
	**diff**	21 (20;23)	31 (29;33)	25 (24;27)	0.83 (-0.19;1.8)	14 (13;15)	16 (15;18)	20 (17;22)	7.7 (6.6;8.7)
	**srm**	0.85 (0.79;0.91)	0.69 (0.63;0.75)	0.95 (0.89;1)	0.041 (-0.01;0.1)	0.6 (0.55;0.66)	0.57 (0.51;0.62)	0.39 (0.34;0.44)	0.37 (0.32;0.43)
2014 (n=1632)	**preop**	38 (37;39)	16 (15;18)	30 (29;30)	61 (60;62)	41 (40;42)	60 (58;61)	44 (42;46)	67 (66;68)
	**year 1**	59 (58;60)	44 (42;46)	53 (52;54)	60 (59;61)	55 (54;56)	75 (74;77)	61 (59;64)	75 (74;76)
	**diff**	21 (19;22)	28 (26;30)	23 (22;25)	-0.44 (-1.5;0.48)	14 (13;15)	16 (14;17)	18 (15;20)	7.3 (6.3;8.4)
	**srm**	0.78 (0.72;0.83)	0.6 (0.55;0.66)	0.84 (0.78;0.89)	-0.02 (-0.07;0.03)	0.58 (0.53;0.63)	0.54 (0.49;0.59)	0.35 (0.3;0.4)	0.35 (0.3;0.4)
2015 (n=1773)	**preop**	38 (37;39)	15 (13;16)	28 (27;28)	60 (59;60)	40 (39;41)	58 (57;60)	42 (40;44)	66 (65;67)
	**year 1**	58 (57;60)	44 (42;46)	52 (51;54)	60 (59;61)	54 (53;55)	75 (73;76)	63 (61;65)	74 (73;75)
	**diff**	20 (19;22)	29 (27;31)	25 (24;26)	0.27 (-0.64;1.2)	14 (13;15)	16 (15;18)	21 (19;24)	7.7 (6.8;8.7)
	**srm**	0.78 (0.72;0.83)	0.64 (0.59;0.69)	0.91 (0.85;0.96)	0.014 (-0.033;0.06)	0.58 (0.53;0.63)	0.56 (0.51;0.61)	0.42 (0.37;0.47)	0.38 (0.33;0.43)
2016 (n=1893)	**preop**	39 (38;40)	15 (13;16)	28 (28;29)	60 (59;61)	40 (39;41)	59 (57;60)	43 (41;45)	66 (65;67)
	**year 1**	60 (59;62)	47 (45;49)	54 (52;55)	61 (60;62)	55 (54;57)	76 (74;77)	65 (63;67)	74 (73;75)
	**diff**	22 (21;23)	33 (31;35)	26 (24;27)	0.68 (-0.2;1.6)	15 (14;16)	17 (16;18)	22 (20;24)	8.3 (7.3;9.2)
	**srm**	0.85 (0.8;0.9)	0.72 (0.67;0.77)	0.93 (0.88;0.99)	0.035 (-0.01;0.08)	0.62 (0.57;0.67)	0.6 (0.55;0.65)	0.44 (0.39;0.49)	0.41 (0.36;0.46)

Table S2c Preoperative and 1-year postoperative SF-36 data (mean and 95% CIs) for patients treated surgically for LDH from 2007 to 2016.

		PF	RP	BP	GH	VT	SF	RE	MH
2007 (n=806)	**preop**	40 (39;42)	9.4 (7.9;11)	23 (22;24)	68 (67;70)	34 (32;35)	48 (46;50)	43 (40;47)	60 (58;61)
	**year 1**	77 (76;78)	62 (60;65)	64 (62;66)	70 (68;72)	60 (58;61)	82 (80;84)	77 (74;79)	77 (76;79)
	**diff**	37 (35;39)	53 (50;56)	41 (39;43)	2 (0.48;3.4)	26 (24;28)	34 (31;36)	33 (30;37)	17 (16;19)
	**srm**	1.4 (1.3;1.5)	1.2 (1.1;1.3)	1.4 (1.3;1.5)	0.099 (0.03;0.17)	0.97 (0.89;1.1)	1 (0.93;1.1)	0.67 (0.59;0.74)	0.81 (0.73;0.89)
2008 (n=883)	**preop**	38 (37;40)	9.6 (8;11)	23 (22;24)	67 (66;68)	35 (33;36)	48 (46;49)	45 (42;48)	61 (59;62)
	**year 1**	76 (74;77)	63 (60;65)	63 (61;64)	69 (67;70)	59 (57;60)	82 (81;84)	78 (76;81)	76 (75;78)
	**diff**	38 (36;39)	53 (50;56)	40 (38;42)	1.9 (0.51;3.3)	24 (22;26)	35 (33;37)	34 (30;37)	15 (14;17)
	**srm**	1.4 (1.3;1.4)	1.1 (1;1.2)	1.3 (1.2;1.4)	0.09 (0.024;0.16)	0.86 (0.79;0.94)	1 (0.96;1.1)	0.65 (0.58;0.73)	0.67 (0.6;0.74)
2009 (n=892)	**preop**	40 (39;42)	10 (8.6;12)	23 (21;24)	67 (66;69)	33 (32;35)	48 (46;50)	45 (42;48)	61 (59;62)
	**year 1**	76 (75;78)	62 (60;65)	63 (61;65)	70 (68;71)	60 (58;61)	82 (80;83)	75 (73;78)	76 (75;78)
	**diff**	36 (34;38)	52 (49;55)	41 (39;43)	2.7 (1.4;4.1)	26 (25;28)	34 (32;36)	30 (27;33)	16 (14;17)
	**srm**	1.3 (1.2;1.4)	1.2 (1.1;1.3)	1.4 (1.3;1.5)	0.14 (0.073;0.2)	1 (0.93;1.1)	1 (0.96;1.1)	0.61 (0.53;0.68)	0.71 (0.64;0.78)
2010 (n=868)	**preop**	40 (38;41)	8.9 (7.5;10)	22 (21;23)	68 (66;69)	33 (32;34)	46 (44;48)	42 (39;45)	60 (59;62)
	**year 1**	77 (75;78)	62 (60;65)	62 (61;64)	70 (68;71)	59 (57;61)	82 (81;84)	77 (75;80)	77 (75;78)
	**diff**	37 (35;39)	53 (51;56)	40 (38;42)	2.1 (0.68;3.5)	26 (24;28)	36 (34;38)	35 (32;39)	17 (15;18)
	**srm**	1.3 (1.2;1.4)	1.2 (1.1;1.3)	1.3 (1.3;1.4)	0.097 (0.03;0.16)	0.97 (0.89;1.1)	1.2 (1.1;1.2)	0.71 (0.64;0.79)	0.72 (0.64;0.79)
2011 (n=890)	**preop**	39 (38;41)	9.8 (8.3;11)	22 (21;23)	66 (65;68)	33 (32;35)	47 (45;49)	43 (40;46)	61 (60;62)
	**year 1**	77 (76;79)	63 (60;66)	63 (61;64)	70 (68;71)	59 (57;61)	83 (81;84)	78 (75;80)	77 (75;78)
	**diff**	38 (36;40)	53 (50;56)	40 (38;42)	3.6 (2.3;5)	26 (24;27)	36 (34;38)	35 (32;38)	16 (14;17)
	**srm**	1.4 (1.3;1.5)	1.2 (1.1;1.3)	1.4 (1.3;1.5)	0.18 (0.11;0.24)	0.99 (0.91;1.1)	1.1 (1;1.2)	0.7 (0.63;0.77)	0.72 (0.64;0.79)
2012 (n=938)	**preop**	39 (37;40)	9 (7.7;11)	22 (21;23)	68 (66;69)	32 (31;34)	47 (46;49)	45 (42;48)	59 (58;60)
	**year 1**	77 (75;78)	64 (61;66)	63 (61;64)	71 (69;72)	59 (58;61)	83 (81;84)	79 (77;82)	77 (75;78)
	**diff**	38 (36;40)	55 (52;57)	41 (39;43)	2.8 (1.5;4.2)	27 (25;28)	35 (33;37)	34 (31;37)	18 (16;19)
	**srm**	1.4 (1.3;1.5)	1.2 (1.1;1.3)	1.4 (1.3;1.4)	0.14 (0.072;0.2)	1 (0.93;1.1)	1.1 (0.99;1.2)	0.65 (0.58;0.72)	0.8 (0.72;0.87)
2013 (n=922)	**preop**	39 (38;41)	10 (8.7;12)	22 (21;23)	67 (66;68)	33 (32;34)	48 (46;49)	44 (41;47)	60 (59;62)
	**year 1**	77 (76;79)	63 (60;65)	62 (60;64)	70 (69;71)	59 (57;61)	83 (81;84)	79 (76;81)	77 (76;78)
	**diff**	38 (36;40)	53 (49;56)	41 (38;42)	2.9 (1.6;4.2)	26 (24;28)	35 (33;37)	35 (32;38)	17 (16;19)
	**srm**	1.4 (1.3;1.5)	1.2 (1.1;1.3)	1.4 (1.3;1.5)	0.15 (0.082;0.21)	1 (0.94;1.1)	1.1 (1;1.2)	0.69 (0.62;0.76)	0.77 (0.69;0.84)
2014 (n=957)	**preop**	41 (39;42)	10 (8.9;12)	23 (22;24)	67 (65;68)	34 (33;35)	48 (46;50)	44 (41;47)	60 (58;61)
	**year 1**	76 (75;77)	61 (58;63)	61 (59;62)	67 (66;69)	58 (56;59)	80 (78;82)	73 (70;76)	76 (74;77)
	**diff**	35 (33;37)	50 (48;53)	38 (36;39)	0.58 (-0.64;1.8)	24 (22;26)	32 (30;34)	29 (26;32)	16 (14;17)
	**srm**	1.3 (1.2;1.4)	1.1 (1;1.2)	1.3 (1.2;1.4)	0.028 (-0.035;0.09)	0.95 (0.87;1)	0.98 (0.9;1.1)	0.57 (0.5;0.63)	0.7 (0.63;0.77)
2015 (n=916)	**preop**	41 (40;43)	10 (9;12)	21 (20;22)	66 (65;67)	33 (32;35)	48 (46;50)	43 (40;46)	61 (60;63)
	**year 1**	76 (74;77)	62 (59;65)	60 (59;62)	67 (66;69)	57 (56;59)	81 (79;82)	75 (72;77)	75 (73;76)
	**diff**	34 (33;36)	52 (49;55)	39 (38;41)	1.6 (0.39;2.9)	24 (22;26)	33 (31;35)	31 (28;35)	14 (12;15)
	**srm**	1.2 (1.2;1.3)	1.1 (1.1;1.2)	1.4 (1.3;1.5)	0.082 (0.017;0.15)	0.94 (0.87;1)	1 (0.95;1.1)	0.6 (0.53;0.67)	0.62 (0.55;0.69)
2016 (n=860)	**preop**	41 (39;42)	9.8 (8.2;11)	20 (19;21)	65 (64;67)	33 (31;34)	46 (44;47)	44 (40;47)	59 (58;60)
	**year 1**	77 (76;79)	62 (59;64)	61 (59;63)	67 (66;69)	58 (56;60)	81 (79;82)	74 (71;76)	75 (74;77)
	**diff**	37 (35;38)	52 (49;55)	40 (39;42)	1.8 (0.49;3)	25 (23;27)	35 (33;38)	30 (27;34)	16 (15;18)
	**srm**	1.4 (1.3;1.5)	1.2 (1.1;1.3)	1.5 (1.4;1.6)	0.092 (0.025;0.16)	0.96 (0.88;1)	1.1 (1;1.2)	0.6 (0.52;0.67)	0.7 (0.63;0.78)

Table S3 Accumulated preoperative and 1-year postoperative SF-36 data (mean and 95% CIs) for patients treated surgically for LSS without DS, LSS with DS, and LDH between 2007 and 2016 and norms for the Swedish general population (Sullivan M, Karlsson J, Taft C, Ware Jr JE. Swedish SF-36 manual and interpretation guide. 2nd ed. Gothenburg: Sahlgrenska University Hospital; 2002).

	**LSS with DS**			**LSS without DS**			**LDH**		
	**Preop**	**Year 1**	**Swe norm[1]**	**Preop**	**Year 1**	**Swe norm[1]**	**Preop**	**Year 1**	**Swe norm[2]**
**PF**	36 (36;37)	59 (58;60)	72 (71;74)	37 (37;37)	58 (57;58)	72 (71;74)	40 (39;40)	77 (76;77)	88 (87;89)
**RP**	13 (12;14)	46 (45;48)	65(62;68)	14 (13;14)	44 (43;44)	65(62;68)	9.8 (9;10)	62 (61;63)	85 (84;87)
**BP**	27 (27;28)	54 (54;55)	69 (67;71)	29 (29;29)	53 (53;54)	69 (67;71)	22 (22;22)	62 (62;63)	73 (72;75)
**GH**	60 (59;60)	62 (62;63)	66 (64;68)	60 (60;60)	60 (60;61)	66 (64;68)	67 (67;67)	69 (69;70)	75 (74;76)
**VT**	39 (38;39)	55 (54;56)	69 (67;71)	40 (40;41)	54 (54;55)	69 (67;71)	33 (33;34)	59 (58;59)	70 (68;71)
**SF**	56 (55;57)	76 (75;76)	86 (85;88)	58 (58;59)	75 (74;75)	86 (85;88)	47 (47;48)	82 (81;82)	88 (87;89)
**RE**	43 (41;44)	65 (63;66)	77 (75;80)	42 (41;43)	62 (61;63)	77 (75;80)	44 (43;45)	77 (76;77)	88 (86;89)
MH	65 (65;66)	75 (74;75)	81 (80;83)	66 (66;66)	74 (74;74)	81 (80;83)	60 (60;61)	76 (76;77)	81 (80;82)

[1] Ages 65-74.
[2] Ages 45-54.

Table S4a Slopes (with 95% CIs) for the regression lines when the trends in SF-36 scores (cf. Fig. S1a) are analysed with linear regression (SF-36 score = a × Year + b where a is the slope and b is the intercept) for patients treated for LSS with DS between 2007 and 2016. Boldface numbers indicate slopes where the 95% CIs exclude zero.

	Preop	Year 1	Difference
PF	**0.52 (0.36;0.68)**	**0.36 (0.059;0.66)**	-0.16 (-0.45;0.12)
RP	**0.42 (0.12;0.71)**	**0.55 (0.11;0.98)**	0.13 (-0.54;0.8)
BP	-0.12 (-0.37;0.13)	**-0.22 (-0.38;-0.055)**	-0.098 (-0.38;0.18)
GH	0.045 (-0.24;0.33)	0.025 (-0.18;0.23)	-0.02 (-0.21;0.17)
VT	0.16 (-0.12;0.43)	-0.15 (-0.44;0.15)	**-0.31 (-0.53;-0.083)**
SF	0.21 (-0.081;0.5)	-0.035 (-0.26;0.2)	-0.25 (-0.66;0.17)
RE	0.48 (-0.16;1.1)	**0.28 (0.0056;0.54)**	-0.2 (-0.85;0.45)
MH	0.16 (-0.011;0.33)	-0.018 (-0.19;0.16)	-0.18 (-0.35;0.0022)

Table S4b Slopes (with 95% CIs) for the regression lines when the trends in SF-36 scores (cf. Fig. S1b) are analysed with linear regression (SF-36 score = a × Year + b where a is the slope and b is the intercept) for patients treated for LSS without DS between 2007 and 2016. Boldface numbers indicate slopes where the 95% CIs exclude zero.

	**Preop**	**Year 1**	**Difference**
**PF**	**0.38 (0.22;0.53)**	**0.41 (0.22;0.6)**	0.036 (-0.1;0.18)
**RP**	0.25 (-0.02;0.52)	**0.49 (0.23;0.75)**	0.24 (-0.1;0.58)
**BP**	-0.086 (-0.26;0.085)	-0.024 (-0.17;0.12)	0.062 (-0.17;0.3)
**GH**	0.041 (-0.084;0.17)	0.052 (-0.11;0.22)	0.011 (-0.096;0.12)
**VT**	0.046 (-0.052;0.14)	0.11 (-0.082;0.3)	0.062 (-0.086;0.21)
**SF**	**0.23 (0.083;0.38)**	**0.14 (0.012;0.27)**	-0.088 (-0.21;0.032)
**RE**	0.18 (-0.11;0.47)	0.3 (-0.031;0.62)	0.12 (-0.22;0.45)
**MH**	**0.2 (0.063;0.35)**	0.094 (-0.072;0.26)	-0.11 (-0.28;0.057)

Table S4c Slopes (with 95% CIs) for the regression lines when the trends in SF-36 scores (cf. Fig. S1c) are analysed with linear regression (SF-36 score = a × Year + b where a is the slope and b is the intercept) for patients treated for LDH between 2007 and 2016. Boldface numbers indicate slopes where the 95% CIs exclude zero.

	**Preop**	**Year 1**	**Difference**
**PF**	0.17 (-0.05;0.39)	0.021 (-0.15;0.19)	-0.15 (-0.46;0.16)
**RP**	0.077 (-0.046;0.2)	-0.097 (-0.3;0.1)	-0.17 (-0.45;0.099)
**BP**	**-0.2 (-0.35;-0.055)**	**-0.35 (-0.51;-0.19)**	-0.14 (-0.39;0.1)
**GH**	**-0.21 (-0.37;-0.046)**	**-0.29 (-0.56;-0.026)**	-0.084 (-0.3;0.13)
**VT**	-0.1 (-0.25;0.054)	**-0.2 (-0.33;-0.066)**	-0.098 (-0.37;0.18)
**SF**	-0.1 (-0.33;0.13)	-0.17 (-0.39;0.043)	-0.071 (-0.44;0.3)
**RE**	-0.033 (-0.33;0.26)	-0.36 (-0.85;0.14)	-0.33 (-0.89;0.24)
**MH**	-0.048 (-0.25;0.16)	**-0.17 (-0.33;-0.0069)**	-0.12 (-0.41;0.17)

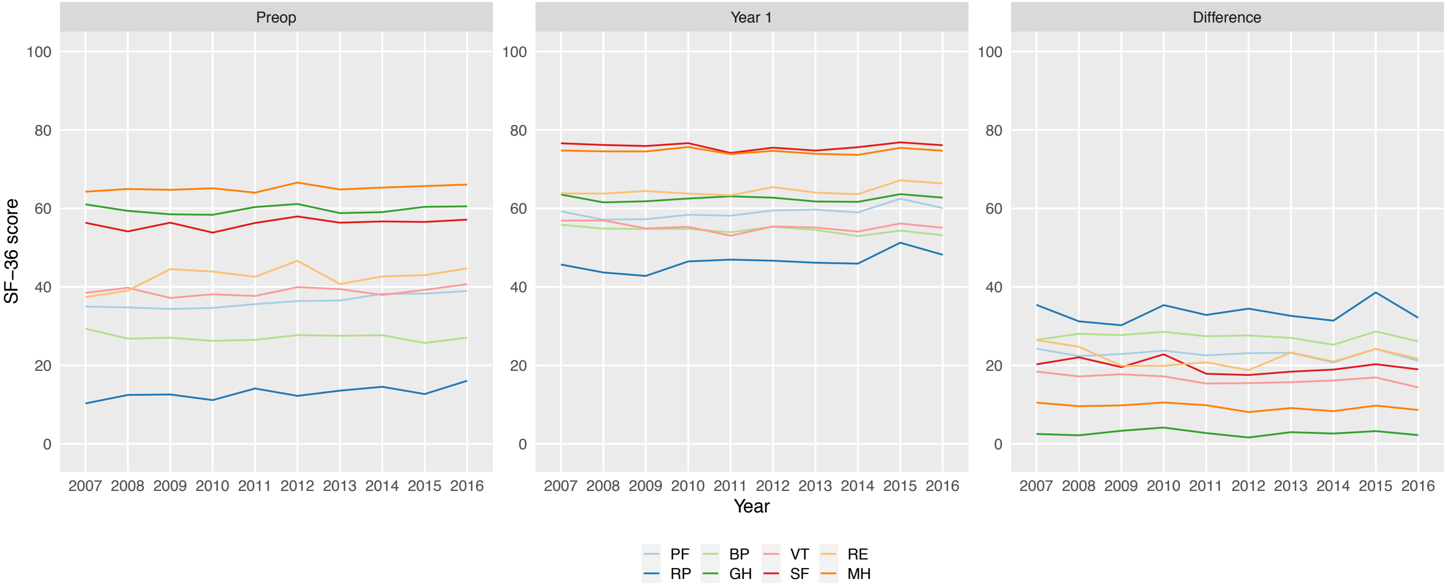

Fig. S1a Trends in SF-36 scores preoperatively and 1-year postoperatively for patients treated for LSS with DS between 2007 and 2016.

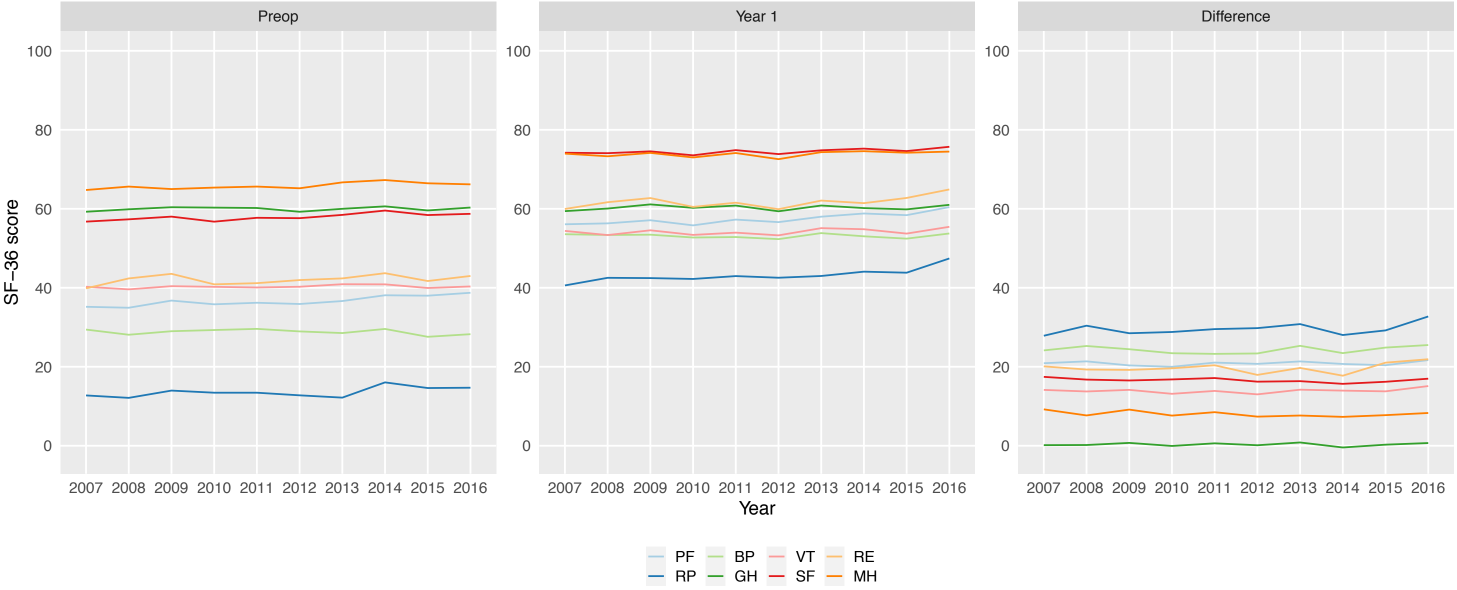

Fig. S1b Trends in SF-36 scores preoperatively and 1-year postoperatively for patients treated for LSS without DS between 2007 and 2016.

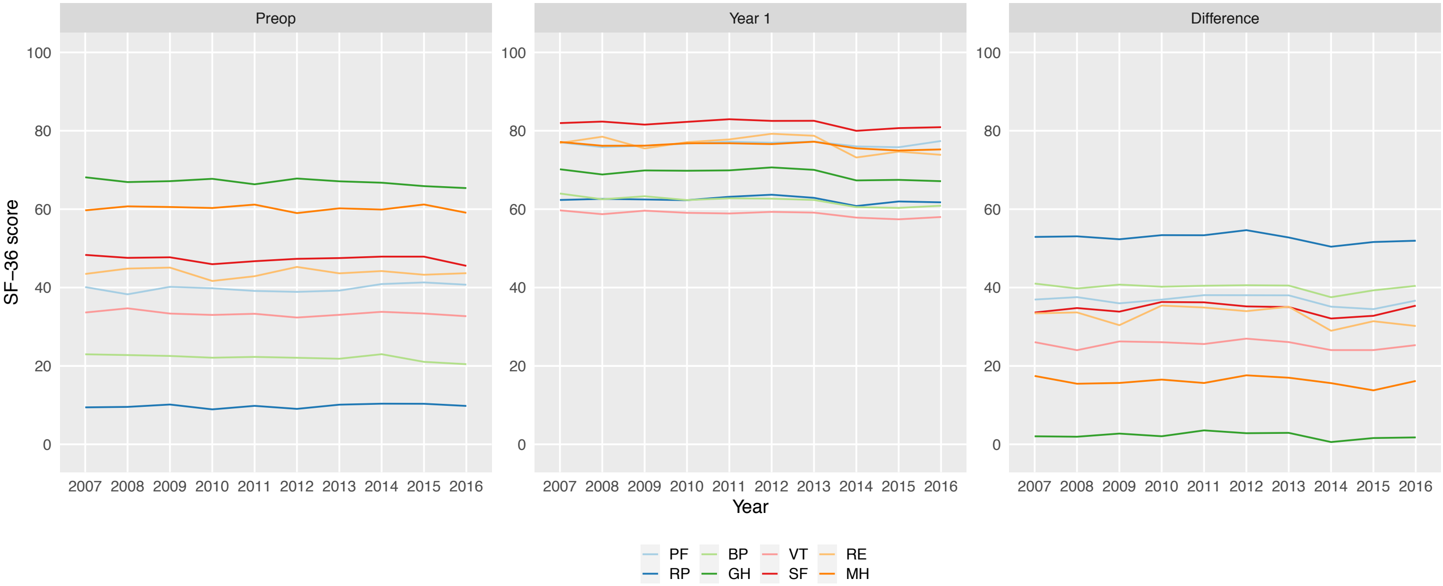

Fig. S1c Trends in SF-36 scores preoperatively and 1-year postoperatively for patients treated for LDH between 2007 and 2016.
